# Decisions about the limitations of life-sustaining treatment for acutely admitted older patients: a retrospective study of a Danish patient cohort

**DOI:** 10.1186/s12877-024-05452-w

**Published:** 2024-10-21

**Authors:** Mathias Eg Lomborg, Jeppe L. C. Nielsen, Skule Arnesen Bakke, Christian Backer-Mogensen, Thomas Strøm

**Affiliations:** 1 Department of Anesthesia and Critical Care Medicine, Odense University Hospital, Svendborg, Denmark; 2 Department of Anesthesia and Critical Care Medicine, Hospital Sønderjylland, Aabenraa, Denmark; 3 Department of Anesthesia and Critical Care Medicine, Lille baelt Hospital, Kolding, Denmark; 4 Department of Regional Health Research, University of Southern Denmark, Odense, Denmark; 5 Department of Emergency Medicine, Hospital Sønderjylland, University Hospital of Southern Denmark, Aabenraa, Denmark; 6 Department of Intensive Care, Odense University Hospital, Odense, Denmark

**Keywords:** End-of-life, Life-sustaining treatment, Patient involvement, Shared decision-making

## Abstract

**Background:**

Life-sustaining therapy, including heart and lung resuscitation and transfer to the intensive care department, is demanding for patients and relatives and utilizes large amounts of healthcare resources. For older patients acutely admitted to the hospital, very sparse data exist on decision making about life-sustaining therapy.

**Methods:**

Retrospective data were extracted from patients ≥ 70 years old who were acutely admitted to the hospital. Age, sex, clinical frailty scale score and Charlson comorbidity index were manually extracted from patients’ files. Furthermore, data about life-sustaining treatment decisions were extracted. This was further divided into decisions documented within 24 h from admission or during the hospital stay.

**Results:**

Data were extracted for 200 patients with a median age of 80 years. Patients had a Charlson Comorbidity Index of 6 (5–8 IQR) and a Clinical Frailty Scale (CFS) score of 5 (3–6 IQR). During the first 24 h, 61 patients (30.5%) had a written decision about heart and cardiopulmonary resuscitation (CPR), and 52 patients (26%) had written information about intensive care therapy. A total of 93 patients (46.5%) had a written decision about cardiopulmonary resuscitation (CPR), intensive care therapy or both during their hospital stay. With increasing Charlson Comorbidity Index and Clinical Frailty Scale scores, more patients had decisions about limitations in therapy documented in their files.

**Conclusions:**

Within the first 24 h, 30.5% of the patients had a written decision about cardiopulmonary resuscitation (CPR), and 26% had written information about intensive care therapy. These numbers increased to 46.5% of patients who had a decision made during their hospital stay whether they were candidates for either cardiopulmonary resuscitation (CPR), intensive care therapy or both. These data suggest that further work should be done to determine the limitations of therapy early on the admission for all older frail acutely admitted patients.

## Background

Advanced life-sustaining therapy has developed in recent years. This became apparent during the COVID-19 pandemic, leading to the consideration of the pros and cons of life-sustaining therapy and considerations of Which patients should be offered cardiopulmonary resuscitation (CPR) attempts or intensive care support. The decision relies on medical, legal and ethical issues.

Patients with a high degree of comorbidities, frailty or old age are at risk of a severe disabilities or death after ICU therapy or CPR. In these cases, CPR or transfer to the ICU should be carefully assessed [1, 2].

However, Life-sustaining therapy should also be in line with patients’ wishes, where personal and religious aspects may be important [3]. This requires that the health professionals try to reveal whether the patients are interested in receiving life-sustaining therapy.

Studies regarding end-of-life practices (EOL) have shown that patients’ wishes for life-sustaining treatment are often unknown. Furthermore, patients’ medical files often lack information on these issues [4–6]. In an acute clinical setting, it might be difficult to clarify patients’ wishes. Therefore, it is important to elucidate these issues in the early or stable stages of the disease. Danish law states that a decision about heart and lung resuscitation should be made when necessary [7]. Furthermore, the covid pandemic made it clear that the healthcare system does not have unlimited resources.

Because of the wide range of consequences for both patients and the health care system of life-sustaining treatment, it is important to examine practices and decision making in a cohort of acutely admitted patients [8].

This study aimed to investigate whether or not a decision was made about CPR or transfer to the intensive care unit would be indicated if needed for frail older patients acutely admitted to the emergency department.

## Method

### Population and setting

Records for patients ≥ 70 years old admitted to the emergency department at Sygehus Sønderjylland from April 2022 to August 2022 were reviewed. This period was chosen to avoid any COVID-19 pandemic interference. Furthermore, only patients acutely admitted with a total time of admission for more than 48 h was included. The hospital has 300 beds. The emergency department receives patients from the following medical specialties: internal medicine, cardiology, gynecology, orthopedic surgery, abdominal surgery, neurology, ear-nose and throat surgery. The hospital is a tertiary hospital, patients need transfer for highly specialized functions (ex. Neurosurgery, thorax surgery or cardiac interventions).

### Conditions

Danish legislation prescribes that a patient can decline life-sustaining treatment, including transfer to the intensive care unit and cardiopulmonary resuscitation. The decision needs to be documented in the patients’ file. Relatives do not have the legal authority to make decisions regarding life-sustaining treatment on behalf of patients who are unable to make these decisions themselves. Physicians make decisions about life-sustaining treatment if the patient wishes are unknown.

### Main outcomes

The main outcome was the proportion of patients with decisions regarding limitations of life-sustaining treatment registered in the patient file. The limitations were “do not resuscitate” in cases of cardiac arrest and “do not transfer to the ICU” in cases of worsening conditions. This was recorded within the first 24 h and during the hospital stay.

The secondary outcomes were the characteristics of patients who were limited by life-sustaining treatment, including the clinical frailty scale and Charlson comorbidity index.

### Ethics approval and consent to participate

This study was conducted in accordance with Danish law, which allows retrospective studies using patient data without individual patient consent when deemed appropriate under specific regulations. According to the Health Act (Sundhedsloven) in Denmark, studies that use de-identified patient data for research purposes do not require written informed consent from the participants.

The need for ethics approval and consent to participate was waived by the Regional Ethics Committee of Region Southern Denmark, as the study involved the retrospective analysis of existing medical records with no direct patient involvement or intervention. Furthermore, this research complies with the General Data Protection Regulation (GDPR) and the Danish Data Protection Act (Databeskyttelsesloven).

As per Danish law, the handling of patient data was approved by the data protection authority, which oversees compliance with data protection regulations. The approval reference number for this study is: Journal nr.: 22/20,240.

### Data registration

The data from patients admitted via the emergency department were extracted from the hospitals’ patient registration system. Date of admission, date of discharge and main allocated specialty were included. Patient characteristics were subsequently manually extracted and constructed from the patient files to a database for 200 patients. The characteristics included the Charlson comorbidity index, clinical frailty scale score, infection with COVID-19, polypharmacy, number of hospital admissions within 12 months up to current admission, death during admission, living arrangements, and decisions regarding life-sustaining treatment (CPR and “do not transfer to ICU”). Furthermore, it was registered whether the decision was made within or after 24 h from admission. The Charlson comorbidity index and clinical frailty scale score was manually and retrospectively constructed according to patients’ files.

The database was pilot tested for feasibility on 20 patients, and minor changes were made. During the data collection, any questions or discrepancies were discussed between the authors before decisions were made. MEL and JCLN made the primary extract of data. Any discrepancies were discussed with TS as senior researcher.

If a patient was admitted more than once during the study period, only data from the first admission were extracted. Patients transferred from other hospitals were also excluded from the data extraction.

### Data analysis

A total of 200 consecutive patients were chosen as a representative number of patients to reflect the decision of life sustaining therapy of patients acutely admitted to the emergency department during the non-COVID-19 period.

All the data are presented with descriptive statistics as medians with interquartile ranges and numbers (%). The data were stratified for decisions made within 24 h or during the hospital stay. Furthermore, the data were stratified by sex, age, polypharmacy, clinical frailty scale score, Charlson comorbidity index score, living conditions and number of hospital admissions.

The chi2 test was used to test between patient stratification and decisions about limitations in life-sustaining treatment. All the data were analyzed using Stata 18 (StataCorp. 2023. Stata Statistical Software: Release 18. College Station, TX: StataCorp LLC). A two-sided p value < 0.05 was considered to indicate statistical significance.

## Results

A total of 200 acutely admitted patients were included. The data were extracted retrospectively from April to August 2022. A median age of 80 years (75–86 IQR) was observed in the patient cohort, and 48.5% of the patients were females (Table 1). Most patients were admitted as medical patients (75%) and a smaller number as surgical patients (25%).

**Table 1:** Baseline data

Total number of patients (*N*)	200
Age (years)	80 (75–86)
Gender	
Female	97 (48.5%)
Male	103 (51.5%)
Type of admission	
Medical	150 (75%)
Surgical	50 (25%)
Polypharmacy*	165 (82.5%)
Positive Covid 19	7 (3,5%)
Living conditions #	
Live at home	90 (45.5%)
Live at home with care	68 (34.5%)
Nursing home	29 (14.5%)
Other	10 (5%)
Charlson Comorbidity Index	6 (5–8)
Clinical Frailty Scale	5 (3–6)
Number of admissions within the last year ¤	
0	61 (31%)
1	70 (36%)
2	36 (18%)
3	15 (8%)
4	9 (8%)
5	2 (1%)
6	1 (0.5%)
7	1 (0.5%)

All values are presented as medians (IQRs) or numbers (%). * Polypharmacy was defined as a daily prescription of more than 5 drugs. # Data for 197 patients. ¤ Data for 195 patients

At baseline, 82.5% of all patients were classified as being receiving polypharmacy treatment with a daily prescription of 5 types or more of medications. A total of 3.5% of the included patients were positive for COVID-19 at admission. Patients had a median Charlson Comorbidity Index of 6 (5–8 IQR) at the time of admission. Furthermore, at admission, patients had a median Clinical Frailty Scale (CFS) score of 5 (3–6 IQR). A total of 45.5% of the included patients were living alone, and 34.5% of patients received care at home. 14.5% of the patients were living at a nursing home at the time of admission. During the last year, patients had a median number of prior admissions of 1 (0–2 IQR) (Table 1).

During the first 24 h after admission, a total of 61 patients (30.5%) had a written decision about heart and cardiopulmonary resuscitation (CPR) made in their patient file (Table 2). A total of 52 patients (26%) had a written information about whether they were candidates for intensive care therapy. Another 31 patients (15.5%) had a decision about whether or not they were candidates for cardiopulmonary resuscitation (CPR) made during their hospital stay; a total of 92 (46%) of all patients had a CPR decision made during their hospital stay. Within 24 h, 52 patients (26%) had a decision about whether or not they were candidates for intensive care therapy. This number increased from an additional 32 patients (16%) during the hospital stay to a total of 84 patients (42%) who had a written decision as to whether they were candidates for intensive care therapy. During the hospital stay, a total of 93 patients (46.5%) had a decision whether they were candidates for heart and cardiopulmonary resuscitation (CPR), intensive care therapy or both (Table 2).

**Table 2:** Decision about limitations in therapy at admission and during the hospital stay

*N* = 200	Within 24 h	During hospital stay	Total during admission
Decision about cardiopulmonary resuscitation (CPR)	61 (30.5%)	31 (15.5%)	92 (46%)
No decision about cardiopulmonary resuscitation (CPR)	139 (69,5%)	108 (54%)	108 (54%)
Decision about intensive care therapy	52 (26%)	32 (16%)	84 (42%)
No decision about intensive care therapy	148 (74%)	116 (58%)	116 (58%)
Decision about cardiopulmonary resuscitation (CPR) or intensive care therapy during hospital stay	62 (31%)	31 (15.5%)	93 (46.5%)
No decision about cardiopulmonary resuscitation (CPR) or intensive care therapy during hospital stay	138 (69%)	31 (15.5%)	107 (53,5%)

All values are presented as numbers (%)

When observing sex or age, no difference was observed in the decision to limit therapy (Table 3). With increasing Charlson Comorbidity Index and Clinical Frailty Scale scores, more patients had a decision about limitations in therapy (12% vs. 1% and 22.5% vs. 12%) (Table 3). As shown in Table 3, no difference in decisions about limitations was observed with respect to living conditions.

**Table 3:** Decision about limitation in therapy by different patient characteristics

	Decision about limitation in therapy *n* = 93 (46.5%)	No decision about limitation in therapy *n* = 107 (53.5%)	*P* value
Gender			
Female	45 (22.5%)	52 (26%)	0.976
Male	48 (24%)	55 (27.5)	
Age (years)			
<80	41 (20.5%)	61 (30.5%)	0.068
>80	52 (26%)	46 (23%)	
Charlson Comorbidity index			
3–5	28 (14%)	65 (32,5%)	
6–9	41 (20,5%)	40 (20%)	< 0.001
10–13	24 (12%)	2 (1%)	
Clinical Frailty Scale			
1–2	4 (2%)	15 (7,5%)	
3–5	44/22%)	68 (34%)	< 0.001
6–9	45 (22,5%)	24 (12%)	
Living conditions #			
Live at home	33 (17%)	57 (29%)	
Live at home with care	37 (19%)	31 (16%)	0.123
Nursing home	16 (8%)	13 (6,5%)	
Other	5 (2,5%)	5 (2,5%)	
Hospital mortality	25 (12.5%)	4 (2%)	< 0.001

# Data for 197 patients

The hospital mortality was 12,5% in the group with limitation compared to 2% in the group with no limitations in therapy during the hospital stay (*P* < 0,001, Table 3).

## Discussion

This retrospective study showed that, during the first 24 h of hospital stay, 31% of patients acutely admitted to the emergency department had a decision made about limitations in therapy with respect to CPR or transfer to the intensive care department. During the total hospital length of stay this number increased to a total of 46.5% of all patients who had a decision about limitation in therapy.

Heart and lung resuscitation and transfer to the intensive care department are demanding tasks, especially for patients and their relatives but also for the healthcare system. Therefore, it is highly important for patients and their relatives to have a discussion on this matter whenever possible and not in an acute setting. This is to best accommodate each patient’s wishes and thoughts. Many patients and relatives have never discussed life sustaining therapy or CPR.

According to Danish law regarding decisions about resuscitation, “The decision must be taken as soon as it is relevant based on a medical assessment. If the patient is seriously ill or dying, the doctor responsible for treatment must assess what should happen in the event of cardiac arrest.” [7].

During the COVID-19 crisis, healthcare resources were potentially even more scarce, and the need for the abovementioned prioritization seemed even more relevant. During the COVID-19 crisis, mortality in Danish intensive care departments differed among regions (30-48.5%) [9]. One explanation for this difference might be differences in the approaches used to address limitations to intensive care therapy during the pandemic. This illustrates the need for early patient involvement and decision making as early as possible after admittance [10]. This discussion is often inconvenient in the acute settings especially for a treating team not knowing the patient.

Jensen et al. conducted a Danish retrospective trial in which patients were acutely admitted with COVID-19 were registered [4]. At the time of admittance, only 16% of the patients had a documented decision about life-sustaining therapy. During admittance, this number increased to a total of 18% of the acutely admitted patients with COVID-19.

A Dutch trial also reported data on Advanced Care planning for patients acutely admitted to the Emergency department for a geriatric evaluation [11]. The authors reported whether patients were referred by a general practitioner or by an elderly care practitioner. Overall, 45.4% of all patients an advanced care plan for life sustaining treatment was known. The percentage was higher when referred by an elderly care practitioner (88.4%) compared to general practitioner 34.8%). The fraction of patients with a known advanced care plan for life sustaining treatment was higher than our data and much higher than the data from Jensen et al. However, patients from the Dutch trial were much more selected with 25% coming from a nursing home and referred by an elderly care physician to a geriatric evaluation.

In the present trial, 46.5% of patients had a decision about life sustaining therapy documented within the first 24 h or during admittance. This was almost twice the percentage of patients seen in the former trial. Furthermore, the former trial included data from the COVID-19 pandemic, during which one could imagine that the need for decisions was even greater. In this light, the decision on life-sustaining therapy reported in the present trial might seem high. On the other hand, one could argue that decisions about life-sustaining therapy should be discussed in all cases of acute or elective admittance, especially in older and fragile patients. The Dutch trial presents a decision on life sustaining treatment of 45.4% at admittance which seems high. But this was much more selected patients referred for an acute geriatric evaluation as mentioned earlier.

Although a referral of an older fragile patient to the emergency department might be the trigger of a discussion about life sustaining treatment, this conversation could also be initiated earlier by the general practitioner. Especially for the frailest patients living at a nursing home. The general practitioner might have a long-term patient relationship and the ability to discuss this in a non-acute situation with the patients and relatives.

Several limitations should be mentioned in the interpretation of the data from the present trial. We only extracted data from 200 patients. This of course holds a risk that our data are not representative and that extracting data from more patients could either increase or decrease our findings. Furthermore, our data were collected from only one emergency department and one hospital. Including more emergency departments and more hospitals, especially from other regions, might have given rise to different numbers.

The findings from the present trial suggest that an effort could be made to discuss the level of treatment for all older acutely admitted patients. This discussion should be made as early as possible, and perhaps it could be an obligatory part when receiving acutely admitted patients.

## Conclusion

In this retrospective trial of 200 older patients acutely admitted to the emergency department within the first 24 h, 30.5% of the patients had a written decision about cardiopulmonary resuscitation (CPR), and 26% had written decision about intensive care therapy. These numbers increased to 46.5% of patients who had a decision made during their hospital stay whether they were candidates for either cardiopulmonary resuscitation (CPR), intensive care therapy or both. These data suggest that further work should be done to determine the limitations of therapy early on the admission for all older frail acutely admitted patients.

## Data Availability

The data that support the findings of this study are available from the authors but restrictions may apply to the availability of these data. Data was used with permission for the current study and so are not publicly available. The data are, however, available from the authors upon reasonable request and with the permission from the hospital administration and the local Danish Data Protection Agency.
